# The mechanisms and roles of melatonin in gastrointestinal cancer

**DOI:** 10.3389/fonc.2022.1066698

**Published:** 2022-12-15

**Authors:** Yong-Qiang Gong, Fu-Tao Hou, Cai-Ling Xiang, Cheng-Long Li, Guo-Huang Hu, Chao-Wu Chen

**Affiliations:** ^1^ Department of Gastrointestinal Surgery, Hunan Provincial People’s Hospital, The First Affiliated Hospital of Hunan Normal University, Changsha, Hunan, China; ^2^ Department of General Surgery, Institute of Digestive Surgery of Changsha, Affiliated Changsha Hospital of Hunan Normal University, Changsha, Hunan, China

**Keywords:** melatonin, gastrointestinal cancer, carcinogenesis, cellular lifecycle, immunity

## Abstract

Gastrointestinal (GI) cancer is a global health problem with wide lesions and numerous cases. The increased morbidity and mortality of GI cancer is a socio-economic challenge for decades to come. Melatonin, a nature indolamine, exerts a crucial role in molecular interactions involved in multiple functional and physiological processes. Increasing evidence indicates that melatonin can modulate GI tract, decrease the occurrence of GI cancer, and enhance the sensitivity to chemoradiotherapy. However, little is known about the exact role of melatonin in anti-carcinogenesis. In this review, we discuss the action of the beneficial effects of melatonin in GI carcinogenesis. Furthermore, we compile the understanding of the role of melatonin in GI cancer, including esophageal cancer (EC), gastric cancer (GC), hepatocellular carcinoma (HCC), colorectal cancer (CRC), and pancreatic cancer (PC). In addition, the potential therapeutic application and clinical evaluation of melatonin in GI cancer are also discussed.

## 1 Introduction

With the development of human civilization and social progress, the average life expectancy has increased significantly. However, cancer is a huge threat to longevity, among which gastrointestinal (GI) cancer is currently one of the major causes of death, with wide lesions and numerous cases. GI cancer includes cancer of the esophagus, stomach, colorectum, liver, and pancreas according to anatomy. It has been reported that colorectal cancer (CRC) is the most fatal and common GI cancer, followed by pancreatic cancer (PC), hepatocellular carcinoma (HCC), gastric cancer (GC) and esophageal cancer (EC) (1, 2). Due to the lack of methods for early diagnosis and effective management, as well as the properties of disease recurrence and metastasis, most GI cancers have a high fatality rate. The efficacy of GI patients has been well improved through surgery, chemotherapy, radiotherapy, etc., but there are still problems in patient management. Therefore, efforts should be made to explore a novel preventative and therapeutic drug therapy to intervene and retard the progress of GI cancer.

**Figure 1:**
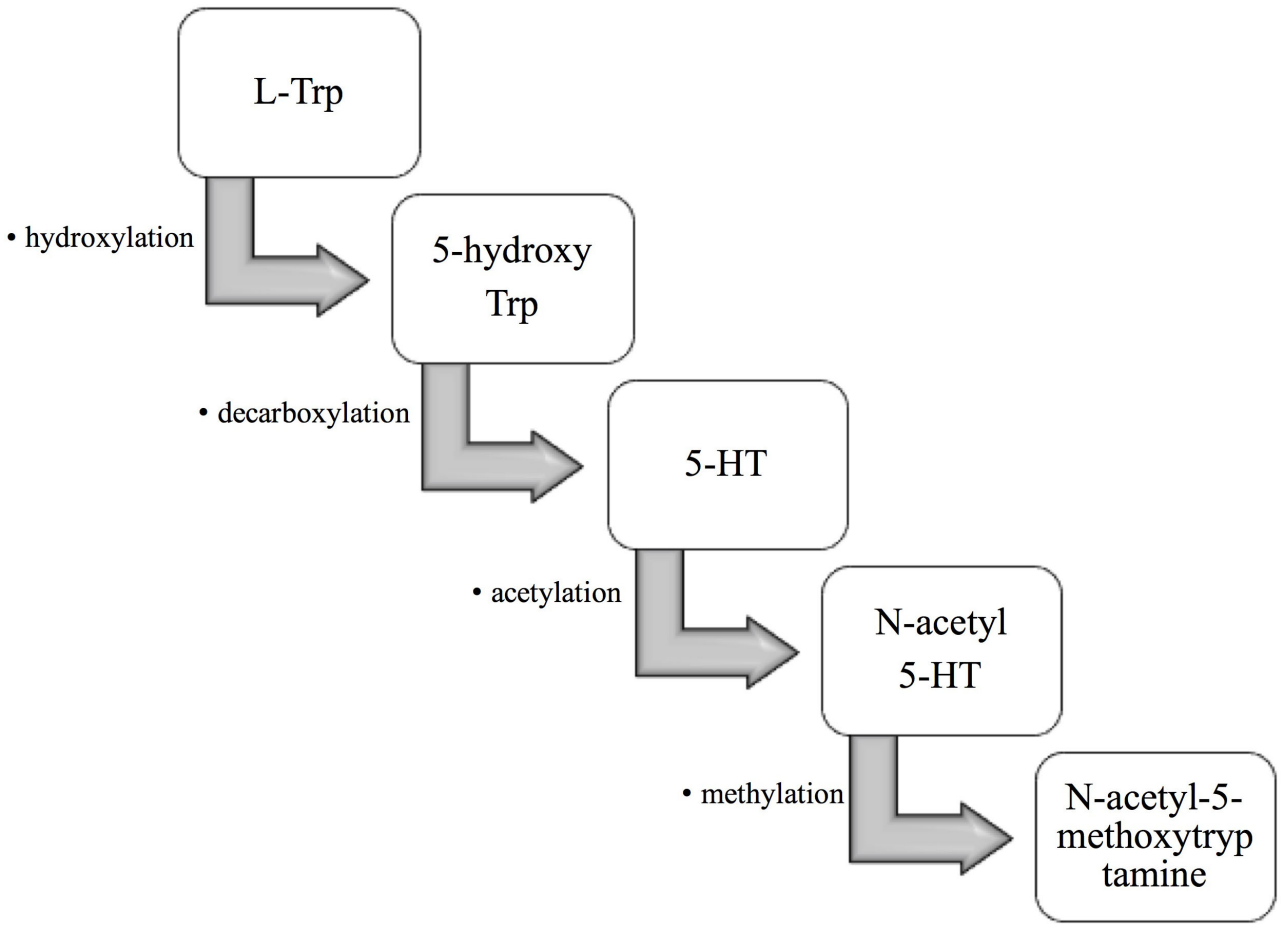
The formation of melatonin in the organism. First, L-tryptophan (L-Trp) is hydroxylated to 5-hydroxytryptophan (5-hydroxy Trp). 5-hydroxy Trp is then decarboxylated to 5-hydroxytryptamine (5-HT), which is then acetylated to N-acetyl-5-hydroxytryptamine (N-acetyl 5-HT). Finally, N-acetyl 5-HT is methylated to N-acetyl-5-methoxytryptamine, also known as melatonin.

L-tryptophan (L-Trp) is hydroxylated to 5-hydroxytryptophan (5-hydroxy Trp), then decarboxylated, acetylated to 5-hydroxytryptamine (5-HT) and N-acetyl-5-hydroxytryptamine (N-acetyl 5-HT), and finally methylated to N-acetyl-5-methoxytryptamine (also known as melatonin) (**Figure 1**). Melatonin is involved in many physiological functions (3, 4). Increasing evidence demonstrates melatonin exhibits antioxidant properties and is responsible for several diseases. It is a strong antioxidant that acts as a scavenger of free radicals (5). Besides, melatonin can activate antioxidant enzymes such as catalase (CAT), glutathione peroxidase (GPX), and superoxide dismutase (SOD), thereby reducing oxidative stress (6). It has been shown that melatonin enhances the expression of antioxidative enzyme genes, protects against depletion caused by ultraviolet radiation-induced, and prevents the formation of DNA damage (6). In addition, melatonin is also known to be an inhibitor of pro-oxidative xanthine oxidase (XO), an activator of DNA repair genes, and protector of mitochondrial membranes, which protect the body from harmful compounds (7). For example, Teixeira et al. indicated that results demonstrate the antioxidant effect of melatonin is mainly corelated with the activities of enzymes such as myeloperoxidase and XO (8). Liu et al. demonstrated that melatonin could increase DNA repair capacity *via* activating genes involved in DNA damage responsive pathways (9).

**Figure 2:**
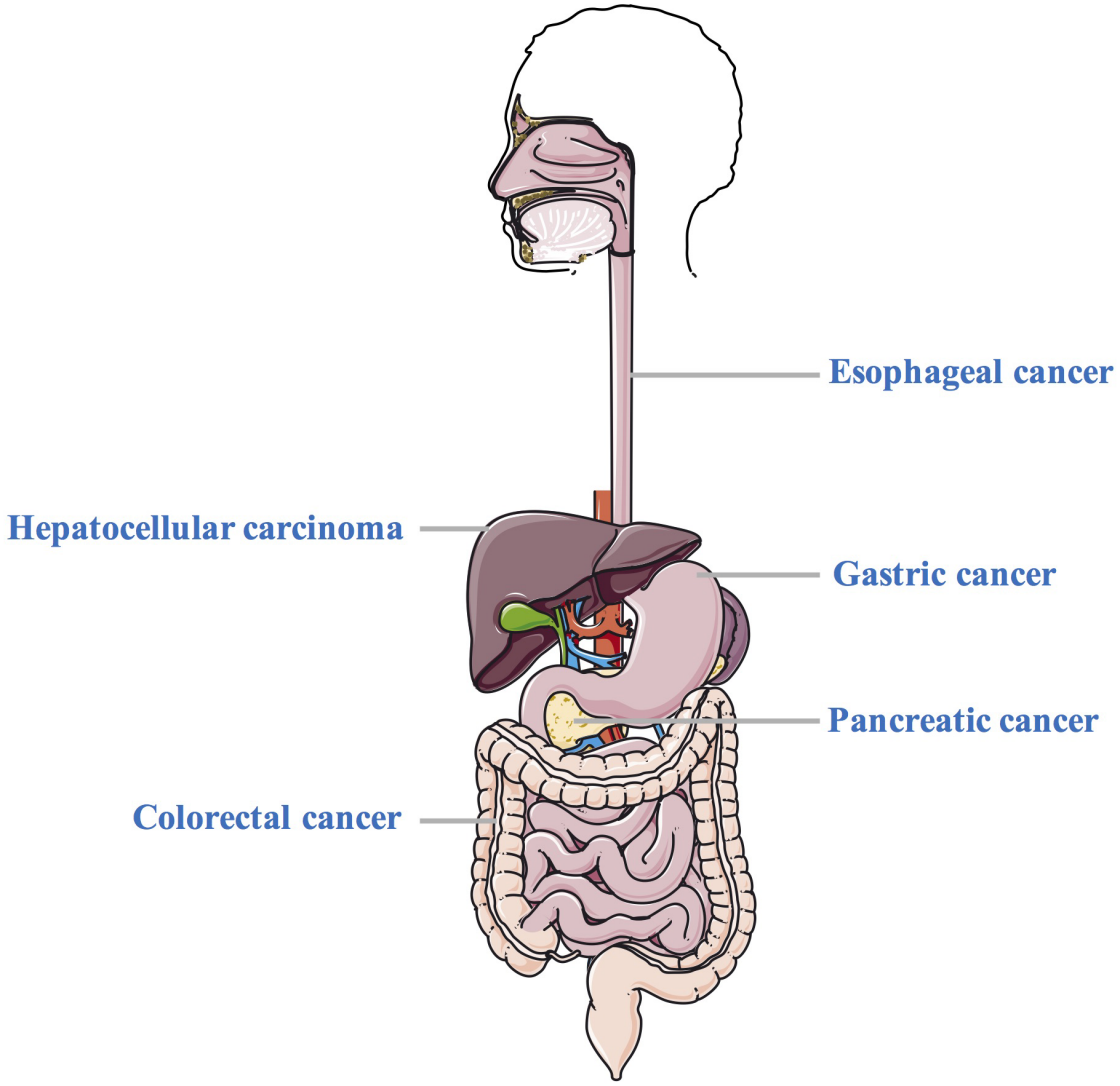
The effects of melatonin on various gastrointestinal cancer. This figure shows the examples of several common gastrointestinal cancer (including EC, GC, HCC, PC, and CRC) where melatonin exhibits protective effects EC, esophageal cancer; GC, gastric cancer; HCC, hepatocellular carcinoma; PC, pancreatic cancer; CRC, colorectal cancer.

Melatonin can be produced by diverse tissues including pineal gland, GI tract, testes, retina, and lymphocytes (10). Melatonin receptors are G-protein coupled receptors, which can be divided into melatonin receptor 1 (MT1) and melatonin receptor 2 (MT2) according to their different affinity (11). A large lines of evidence have indicated that melatonin is a vital regulator of circadian and seasonal rhythms (12, 13). Given that melatonin is a “jack-of-all-grades”, it is not surprising that it affects the progression of GI cancer. For example, Parent et al. demonstrated that night shifts may affect cancer risk by inhibiting melatonin release (14). Wang et al. confirmed that melatonin is associated with the GC metastasis and poor prognosis (15). However, the mechanisms and roles by which melatonin can modulate the GI carcinogenesis are elusive. Therefore, we compile the research progress of melatonin and GI cancer (**Figure 2**), including EC, GC, HCC, PC, and CRC, to provide theoretical basis and ideas for further revealing melatonin and GI cancer. Moreover, the potential therapeutic application and clinical evaluation of melatonin in GI cancer are also discussed.

## 2 The mechanism of action of melatonin in GI carcinogenesis

**Figure 3:**
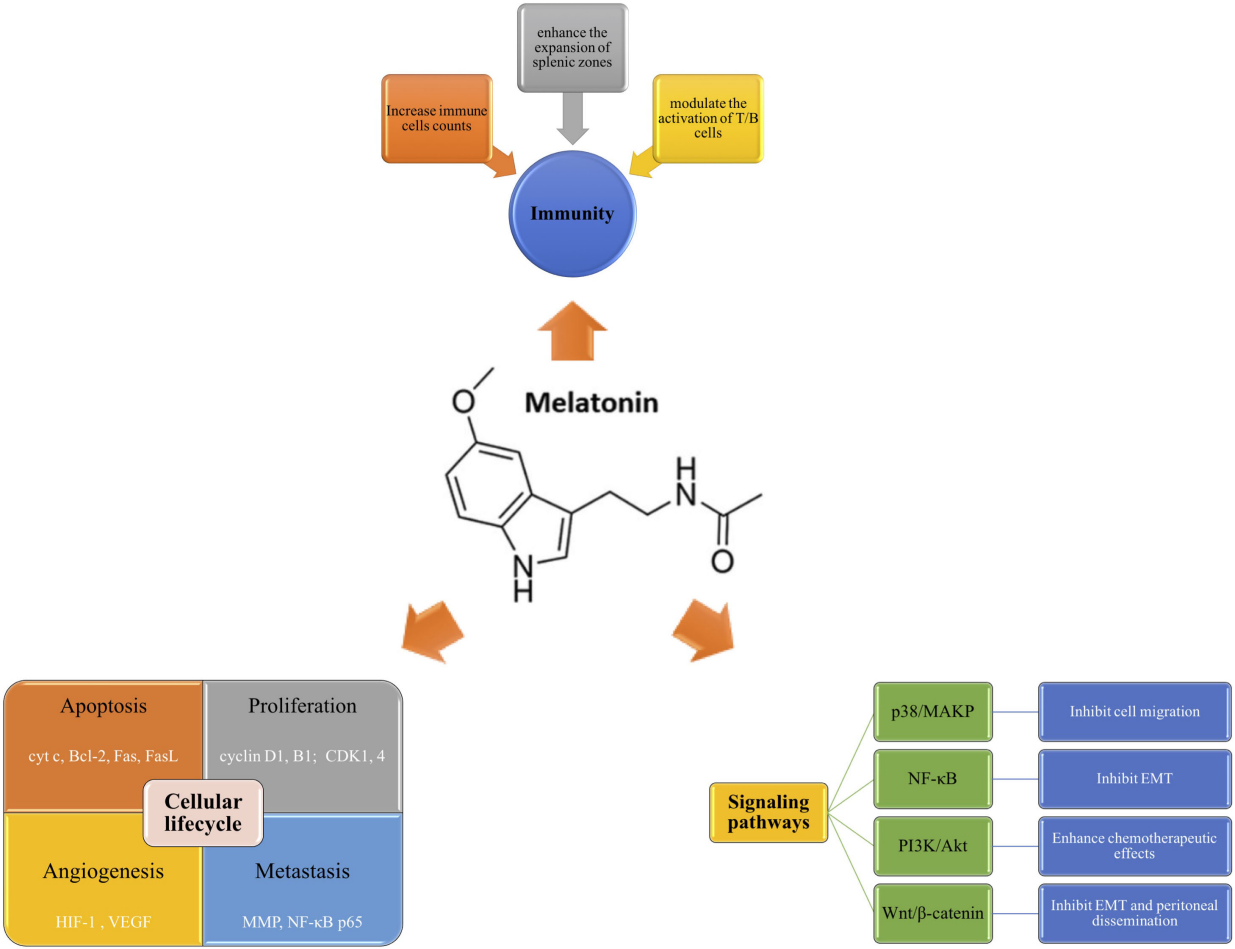
Mechanisms of melatonin in GI carcinogenesis. Melatonin plays a role in anti-carcinogenesis mainly through the following ways, including modulating cellular lifecycle, regulating immunity function, and involving several oncogenic signaling pathways. Melatonin can induce cell apoptosis *via* regulating multiple genes (cyt c, Bcl-2, Fas) and inhibit proliferation by arresting cancer cell cycle (cyclin D1, cyclin B1, CDK1, CDK 4). Moreover, it can also influence the angiogenesis and metastasis by modulating HIF-1, VEGF, MMP, etc. Secondly, melatonin is a regulator of immunity. It mediate the immune function mainly through increasing the counts of immune cells, enhancing the expansion of splenic zones, and activating the function of T/B cells. Thirdly, melatonin can inhibit carcinogenesis through specific signaling pathways, such as p38/MAPK, NF-κB, PI3K/Akt, and Wnt/β-catenin. Bcl-2, B-cell lymphoma-2; cyt c, cytochrome c; EMT, epithelial-mesenchymal transition; FasL, Fas ligand; HIF-1, hypoxia-inducible factor 1; MAPK, mitogen-activated protein kinase; MMP, matrix metalloproteinases; NF-κB, nuclear factor-kappa B; PI3K, phosphoinositide 3 Kinase; VEGF, vascular endothelial growth factor.

Circadian rhythm disturbance is closely related to the occurrence and development of cancer. Studies have shown that melatonin, normally up-regulated at night, helps to stabilize metabolic rhythm, thus involving cancer progression (16, 17). Melatonin exerts its anti-carcinogenesis role through various ways, including promoting cancer cell apoptosis, inhibiting proliferation, regulating angiogenesis and metastasis, modulating immunity, and involving several oncogenic signaling pathways (**Figure 3**). However, the intracellular signaling pathway of melatonin has not been clearly defined.

### 2.1 Melatonin and cellular lifecycle

Cell proliferation, differentiation, senescence and apoptosis are structural and functional bases of organism growth, development, aging and death, respectively (18). Cells deviate from the normal lifecycle due to internal or external factors, which may lead to the occurrence of cancer. It has been reported that melatonin act as a loyal defender against GI cancer through regulating cellular apoptosis, proliferation, metastasis, and angiogenesis. With the relevant cumulative findings, herein we investigate the action and role of melatonin in GI carcinogenesis.

Apoptosis, also known as programmed cell death (PCD), is a physiologic cell death that is distinct from necrosis. It is an active “suicide” extinction process after cells are stimulated by certain signals. Recent in-depth studies on biological cell death pathways have shown that the attenuation of apoptosis is closely related to the formation of GI cancer (19). There is increasing evidence that melatonin promotes PCD in GI cancer (20). Cytochrome c (cyt c) is release into the cytoplasm when the cells are stimulated, and triggers an enzymatic cascade that leads to apoptosis (21). The B-cell lymphoma-2 (Bcl-2) family mediates the intrinsic apoptosis pathway with both anti-apoptotic and pro-apoptotic effects (22). Fas, a transmembrane protein, binds to Fas ligand (FasL) to initiate the transduction of apoptotic signals and induce apoptosis (23). Mechanistically, melatonin can modulate the expression of multiple genes associated with apoptosis, such as cytosolic cyt c, Bcl-2, Fas, etc (20, 24).

Rampant proliferation is another important characteristics of all cancers. The carcinogenesis is the result of the imbalance between cell proliferation and apoptosis. It has been reported that melatonin exerts an obvious anti-proliferation effect *via* arresting cancer cell cycle (25). For example, Liu et al. proved that melatonin attenuated the expression of cyclin D1 and CDK4 in G1 phase, and cyclin B1 and CDK1 in G2/M phase of human osteosarcoma cells (26). Moreover, the anti-proliferative efficacy of melatonin have been demonstrated in GC and HCC cell lines (27).

Additionally, increasing evidence indicates that melatonin is also involved in angiogenesis and metastasis in GI cancer. Cancer angiogenesis is known to be an important feature of metastasis responsible for cancer death. Melatonin has the capacity to reduce the migration and neovascularization of cancer cells. For instance, Wang et al. showed that melatonin suppressed IL-1β-induced lung metastasis of GC by downregulating the expression of matrix metalloproteinases (MMP), and nuclear factor-kappa B (NF-κB) p65 (28). Moreover, melatonin can also inhibits cancer angiogenesis *via* attenuating hypoxia-inducible factor (HIF)-1 and decreasing vascular endothelial growth factor (VEGF) expression (29).

### 2.2 Melatonin and immune function

Interactions between immune cells and cancer cells exert a crucial role in cancer development (30). Since MT are widely present in immune system, melatonin is involved in regulating immune function. Studies have shown that melatonin increases the cells of the innate immunity, such as neutrophil, macrophages, and lymphocytes counts (31). Luo et al. demonstrated that melatonin modulated the activation of T/B cells, thus playing a key role in stabilizing immune balance (32). Besides, Liu et al. indicated that melatonin inhibited GC cell growth by down-regulating the expressions of CD4 (+) and CD25 (+) regulatory T cells (Tregs) and Forkhead box p3 (Foxp3) in GC (33). Moreover, melatonin is also proved to enhance the expansion of splenic zones (34). In addition, it has been shown that melatonin participates in the regulation of cytokine production (35).

### 2.3 Melatonin and signaling pathways

Signaling pathways are intertwined into networks in physiological processes of systemic organs throughout the body and play important roles in human health and disease. The disruption of multiple signaling pathways is closely involved in the progression of diverse cancers. It has been indicated that melatonin could regulate the mediators in oncogenic signaling pathways, such as NF-κB, phosphoinositide 3 Kinase (PI3K)/Akt, p38/mitogen-activated protein kinase (MAPK), and Wnt/β-catenin axis (36–38). For example, Liu et al. reported that melatonin decreased Rho−associated protein kinase (ROCK) expression *via* p38/MAPK signaling pathway, thus inhibiting the migration of CRC cells (39). The transdifferentiation of epithelial cells into motile mesenchymal cells, known as epithelial-mesenchymal transition (EMT), is a process that enhances the invasiveness and anti-apoptotic capabilities of cancer cells (40). Wang et al. proved that melatonin suppressed EMT in GC *via* attenuation of IL−1β/NF−κB/MMP2/MMP9 axis (41).

## 3 The role of melatonin in GI cancer

Despite tremendous scientific breakthroughs in understanding the mechanistic properties of GI cancer, therapeutic efficacy remains very limited. Increasing evidence demonstrates that melatonin has positive protective effects against both endogenous stimuli (acid and pepsin) and exogenous insults (alcohol and stress) affecting the GI tract (42). There are numerous studies assessing the roles of melatonin on health and disease. Melatonin has been seen as an adjunctive treatment for advanced cancer because of its anti-inflammatory and anti-oxidant effects. In this part, we discuss the role of melatonin in GI cancer, including EC, GC, HCC, CRC, and PC, respectively (**Table 1**).

**Table 1:** Effects of melatonin against various gastrointestinal cancer.

Cancer types	Targets	Effects	Ref(s)
Esophageal cancer	Erk, Akt	improve ESCC cell sensitivity to 5-FU	(43, 44)
	EZH2	suppress EC cell proliferation and migration	(45)
	NF-κB	exert anti-inflammatory and anti-oxidant roles	(46)
	PI3K/Akt	induce cell apoptosis and cell cycle arrest	(47)
Gastric cancer	ROS, MMP2, MMP9	inhibit GC cell growth	(48)
	miR-15-5p-Smad3	suppress GC cell proliferation	(49)
	NF-kB and MAPK	induce GC cell apoptosis	(50)
	VEGF, Wnt/β-catenin	regulate angiogenesis and differentiation	(51)
	MMP2, MMP9, NF-kB p65	reduce lung metastasis	(28)
	NF-κB, C/EBPβ	block EMT and peritoneal dissemination	(52)
Hepatocellular carcinoma	ER	induce HCC cell apoptosis	(53)
	transcription factors	inhibit HCC cell proliferation and invasiveness	(54)
	ncRNAs	restrain HCC progression	(55, 56)
	RAD51-AS1	sensitize HCC cell to chemotherapy	(57)
	mTORC1	suppresses glycolysis	(58)
Colorectal cancer	miR-34a/449a cluster	regulate cell cycle	(59)
	ER	promote CRC cell apoptosis	(60)
	XIAP	increase 5-FU-mediated apoptosis	(61)
	/	sensitize CRC cell to γ-ray ionizing radiation	(62)
	lipid metabolism, gut microbiota	play a preventive and therapeutic role in CRC	(63)
Pancreatic cancer	VEGF, HSPs, caspase-3, caspase-9	stimulate PC cell apoptosis	(64, 65)
	PDGFR-β/STAT3	enhance the efficacy of chemotherapy	(66)

### 3.1 The role of melatonin in esophageal cancer

Esophageal cancer (EC) refers to malignant tumors of esophageal epithelial origin, which is mainly manifested as choking sensation when swallowing food, foreign body sensation, retrosternal pain, or obvious dysphagia (67). It is one of the most common gastrointestinal cancer with regional variations in morbidity and mortality. The two major subtypes of EC are esophageal squamous cell carcinoma (ESCC) and esophageal adenocarcinoma (EAC). Studies have shown that smoking, alcohol consumption, obesity, aging, male sex, Barrett’s esophagus, and gastroesophageal reflux disease (GERD) consist of the potential risk factors for EC (68, 69).

Melatonin has been shown to afford esophago-protection *via* diverse mechanisms. Firstly, it enhances tumor cell sensitivity to chemotherapy drugs. In 2016, Lu et al. demonstrated that melatonin improved ESCC cell sensitivity to 5-fluorouracil (5-FU) *via* suppressing the Erk and Akt pathway (43). Furthermore, Zhang et al. proved that melatonin significantly enhanced 5-FU-mediated suppression of esophageal cancer cell proliferation and migration by regulating enhancer of zeste homolog 2 (EZH2) (45). Histone methyltransferase EZH2, a catalytic component of polycomb repressive complex 2 (PRC2), is highly expressed in the development of esophageal cancer (70). Secondly, melatonin exerts anti-inflammatory and anti-oxidant effects in EC. NF-κB induces the expression of multiple genes through the activation of stimulating factors and produces multiple cytokines involved in inflammatory responses. Gu et al. revealed that melatonin inhibited cell invasion *via* down-regulating the NF-κB signaling pathway in EC (46). Thirdly, melatonin is involved in the regulation of cell cycle. It has been reported that melatonin causes cell cycle arrest and affects the percentage of abnormalities in different phase (47).

### 3.2 The role of melatonin in gastric cancer

Gastric cancer (GC) is a predominant malignancy with the second leading cause of cancer death worldwide (71). The lifestyle, genetics, helicobacter pylori (H. pylori) infection, and epigenetics are potential risk factors for GC (72). H. pylori is a major cause of gastric carcinogenesis by enhancing the formation of reactive oxygen species (ROS) and reactive nitrogen species (RNS), and inducing local ulceration and inflammation (73). According to the pathological types, GC can be divided into adenocarcinoma, signet ring cell carcinoma, adenosquamous carcinoma, medullary carcinoma and undifferentiated cell carcinoma, among which adenocarcinoma is the most common. Gastroscopy is the preferred method for GC, and gastric biopsy is the “gold standard” for the diagnosis of GC.

Growing evidence demonstrates that melatonin exerts gastroprotective effects in GC through multiple mechanisms, including increasing blood flow, reducing inflammation, scavenging free radicals, and inhibiting MMP (41, 74, 75). On the one hand, melatonin has been shown to play a role in affecting the growth of GC itself. Liu et al. revealed that melatonin inhibited GC cell progression *via* the NF-kB signaling pathway (48). They proved that melatonin suppressed cell growth by directly reducing ROS production, while indirectly decreasing the level of MMP2 and MMP9 in cancer-associated fibroblasts (CAFs) in gastric cancer cells. Moreover, melatonin was reported to inhibit the proliferation of GC cells *via* modulating miR-15-5p-small mothers against decapentaplegic homolog 3 (Smad3) pathway (49). Additionally, it has been demonstrated that melatonin induces GC cell apoptosis *via* regulating diverse signaling pathways (76, 77). For example, Li et al. noted that melatonin stimulated GC cell apoptosis by mediating NF-kB and MAPK signaling pathways (50). Apart from mentioned above, the effects of melatonin on angiogenesis and differentiation are also involved in the progression of GC (51). On the other hand, melatonin has also been proven to be effective in reducing metastasis exacerbations. There is increasing evidence that GC migration is significantly reduced following melatonin treatment. In a study, decreased lung metastasis in GC after melatonin treatment was associated with the downregulation of MMP2, MMP9, and NF-kB p65 (28). Wu et al. indicated that melatonin blocked EMT and peritoneal dissemination through NF-κB cleavage and calpin-mediated C/EBPβ (52). Besides, melatonin was reported to inhibit the migration of GC *via* remodeling tight junction (78).

### 3.3 The role of melatonin in hepatocellular carcinoma

Hepatocellular carcinoma (HCC) is a major global health challenge and the fourth leading cause of cancer-related death worldwide. HCC is prevalent to spread in the liver through the portal vein system, forming intrahepatic metastasis, and also easy to form tumor thrombus in the portal vein, causing the manifestation of portal hypertension (79). A commonly used tumor marker for HCC is alpha-fetoprotein (AFP), especially when it is significantly elevated, high vigilance should be exercised. For people with hepatitis infection, liver cirrhosis and family history of liver cancer, regular screening for HCC should be carried out for early detection, diagnosis and treatment (80).

Emerging evidence demonstrates that melatonin exerts anti-cancer activity on HCC. It is worth noting that melatonin reverses apoptosis resistance and activates both intrinsic and extrinsic pathways of apoptosis in HCC (54). Zha et al. found that melatonin stimulates endoplasmic reticulum (ER) stress-induced apoptosis in HCC (53). Moreover, melatonin is involved in the regulation of HCC by modulating a variety of transcription factors and related pathways to inhibit cell proliferation and invasiveness (54). In addition, melatonin has been proven to restrain HCC progression *via* regulating non-coding RNAs (ncRNAs), such as microRNAs (miRNAs) and long non-coding RNAs (lncRNAs) (55, 56). Besides, melatonin could sensitize HCC cell to chemotherapy (57). Recent studies have shown that melatonin suppresses glycolysis in HCC cells by down-regulating mitochondrial respiration and mammalian target of rapamycin complex 1 (mTORC1) activity (58).

### 3.4 The role of melatonin in colorectal cancer

Colorectal cancer (CRC) is considered as the second most common cancer worldwide and the incidence of CRC increases with age (81). Adenocarcinoma is the most common pathological type of CRC. In recent years, the morbidity and mortality of colorectal cancer have shown an increasing trend, which should be paid more attention to. Chemoradiotherapy is the last-line treatment for advanced CRC with significant side effects. Therefore, it is urgent to explore an anti-cancer agent in CRC.

Melatonin holds promise as an adjunctive treatment for advanced CRC. The entero-endocrine (EE) cells in GI tract mucosa are the main source of intestinal melatonin (82). Increasing evidence evaluates the effects and safety of melatonin. It has been reported that melatonin is associated with various health outcomes in heterogeneous populations (83). Mechanically, it has been reported that melatonin prevent and delay the progression of CRC by suppressing the proliferation and inducing apoptosis of CRC cells. Ji et al. demonstrated that melatonin regulated the miR-34a/449a cluster, thus influencing the cell cycle in CRC (59). Yun et al. noted that melatonin promoted CRC cell apoptosis *via* superoxide-mediated ER stress (60). Besides, melatonin promotes chemotherapeutic drug-mediated apoptosis of CRC cells by enhancing oxidative stress (61). In addition to its role in sensitivity to chemotherapeutic agents, melatonin also sensitizes human CRC cells to γ-ray ionizing radiation both *in vitro* and *in vivo* (62). Interestingly, studies have pointed out that melatonin plays a preventive and therapeutic role in CRC by regulating lipid metabolism and gut microbiota (63). Menadione, a synthetic form of vitamin K, is known to stimulate an increase in intracellular ROS and alter the oxidative status of cancer cells (84). Collin et al. confirmed that menadione plus melatonin on Caco-2 cells could reduce cell proliferation, induce reactive nitrogen species formation, enhance superoxide anion content, and increase catalase activity, suggesting the potential as adjuvant therapy for CRC acting on different oncogenic pathways (85). Kvietkauskas et al. designed a study to explore the combined role of melatonin and glycine in CRC liver metastasis (86). They found that supplementation with melatonin and glycine reduce CRC liver metastasis growth by acting as natural antiangiogenic molecules.

### 3.5 The role of melatonin in pancreatic cancer

Pancreatic cancer (PC) is the source of an increasing number of cancer-related deaths with a low survival rate. The high mortality rate of PC is likely to be associated with the absence of early symptoms and therefore delayed diagnosis, as well as high resistance to chemoradiotherapy. PC is classified as resectable, borderline, locally advanced and metastatic with greatly varied treatment among them (87). The sensitivity of computed tomography (CT) and magnetic resonance imaging (MRI) in detecting PC was as high as 96% and 93.5%, respectively (88). Despite significant advances in the treatment of PC, including improved surgical techniques and refined adjuvant and neoadjuvant therapies, the incidence and mortality of PC have not decreased significantly worldwide. We therefore emphasize the historical perspective of PC treatment, highlight the prevention strategies, and identify more integrated approaches.

Growing evidence supports the therapeutic benefits of melatonin in the management of PC. Studies have shown that a variety of inflammatory pathways are closely related to the pathological process of PC (89). Melatonin stimulates cancer cell apoptosis by acting as an inflammatory inhibitor, an oxidative stress modulator, a VEGF inhibitor, a heat shock proteins (HSPs) inhibitor, etc. (64, 65). Firstly, it protects pancreatic tissue from inflammatory damage and oxidative stress *via* activating anti-oxidant enzymes and scavenging ROS and RNS. Secondly, melatonin decreases endothelial cells proliferation and reduces angiogenesis by inhibiting VEGF. Thirdly, high concentration of melatonin regulate Bax/Bcl protein balance, thus stimulating the expression of caspase-3 and caspase-9, while low level of melatonin produce anti-apoptotic HSPs, such as HSP27 and HSP90, thereby preventing the activation of caspase-3. Moreover, it has been found that melatonin is involved in enhancing the efficacy of chemotherapy and decreasing side effects. Fang et al. found that melatonin and sorafenib synergistically inhibited PC through MR and PDGFR-β/STAT3 signal pathway (66). Melatonin was also proved to enhance the chemosensitivity to gemcitabine in PC (90). The metabolites of L-Trp and melatonin are called kynuramines, of which N1-acetyl-5-methoxy-kynuramine (AMK) and N1-acetyl-N1-formyl-5-methoxykynuramine (AFMK) are the best known melatonin derivatives (91). It has been shown that melatonin precursor L-Trp and the melatonin derivatives kynuramines, may be related to the physiological and functional failure of the pancreas, leading to the impairment of pancreatic function and anti-cancer ability (92).

## 4 The safety evaluation of melatonin in GI cancer

Melatonin is an endogenous molecule that has its own metabolic pathway in the body. The biological half-life is short and falls to the physiological level of normal after 7~8 hours of oral administration. Therefore, it is a relatively safe substance for clinical use in humans. The underlying mechanisms of melatonin *in vitro*, such as cell apoptosis, cell proliferation, immune function, and signaling pathways, have largely been validated *in vivo* studies. For clinical application, a large number of researches have further examined the role of melatonin in GI cancer *in vivo*. On the one hand, many studies have explored the effects of melatonin on GI cancer in animal models. For example, Winczyk et al. constructed an animal model of colon cancer in mice (93). They found an increase of apoptotic cells in cancers treated with melatonin, further confirming the pro-apoptotic efficacy of melatonin on murine colon cancer cells. On the other hand, the clinical trials have also been conducted to evaluate the safety and efficacy of melatonin in GI cancer. It is well known that melatonin’s sleep-inducing effects have been widely used clinically (94). Recent clinical studies have shown that melatonin can improve the survival rate of patients with GI cancers, increase the sensitivity to chemotherapeutic agents, and reduce the side effects of chemoradiotherapy (38). For example, Kouhi et al. conducted a double-blind controlled study in 60 patients with rectal cancer, in which the experimental group received 20 mg melatonin a day and the control group treated with placebo (95). Their subsequent study found that radiotherapy induced less severe reductions in blood cell counts in patients treated with melatonin, suggesting a role for melatonin in reversing the adverse effects of radiation. The above studies all indicate that melatonin can be utilized for the treatment of GI cancer. However, the appropriate dosage and optimal duration of melatonin still require extensive studies to specifically validate the effects of melatonin on GI cancer progression in the future.

## 5 Conclusions and perspectives

Melatonin, a natural indolamine, is produced by a variety of tissues and is involved in the mediation of physiological functions. Given that MT are widely distributed in many organs and tissues of the body, it is not surprising that it is called a “jack-of-all-grades”. GI cells can not only secrete melatonin, but also have MT on them, thus exerting important protective and regulatory roles. Studies have shown that melatonin boosts the GI immune system, regulates fecal moisture, slows intestinal peristalsis, and protects the GI tract from digestive enzymes and stomach acid. Emerging evidence proves that melatonin affects the progression of GI cancer. Despite our understanding of how melatonin exerts its anti-cancer effects is expanding, much remains to be studied. There are many challenges in translation to therapeutic applications in GI cancer, such as safety assessment and bioavailability. Future researches should continue to focus on the communication between melatonin metabolism and the development and progression of GI cancer.

## Author contributions

Y-QG collected literature and wrote the manuscript. F-TH summarized the table and drew the figures. C-LX and C-LL supervised the manuscript and modified the figures. G-HH and C-WC conceived the idea and supervised the manuscript. All authors read and approved the final manuscript.
